# Initial and long-term evaluation of patients with Alzheimer’s after hospitalization in cognitive and behavioural units: the EVITAL study design

**DOI:** 10.1186/s12888-014-0308-6

**Published:** 2014-11-15

**Authors:** Elodie Pongan, Magalie Freulon, Floriane Delphin-Combe, Florence Dibie-Racoupeau, Géraldine Martin-Gaujard, Denis Federico, Aziza Waissi, Gaëlle Richard, Sophie Jacqueline, Florence Fabre, Béatrice Trombert-Paviot, Pierre Krolak-Salmon, Bernard Laurent, Isabelle Rouch

**Affiliations:** Service de Neurologie, CM2R, CHU de Saint Etienne, Hôpital Nord, Avenue Albert Raimond, 42055 Saint Etienne, France; Hospices civils de Lyon, CM2R, Hôpital gériatrique des Charpennes, 27 rue Gabriel Péri, 69100 Villeurbanne, France; Centre hospitalier St Jean de Dieu, 290 Route de Vienne, 69008 Lyon, France; Service de Gérontologie clinique, CM2R, CHU de Saint Etienne, Hôpital Nord, Avenue Albert Raimond, 42055 Saint Etienne, France; Public Health and Medical Informatics unit, University hospital of Saint-Etienne, 42055 Saint Etienne, France; EA 4607 SNA- EPIS PRES Lyon, Université Jean Monnet, 42023 Saint Etienne cedex, France; Dynamique Cérébrale et Cognition INSERM U1028 - CNRS UMR5292, Centre de Recherche en Neurosciences de Lyon, 95 boulevard Pinel, 69500 Bron, France

**Keywords:** Alzheimer’s disease and related disorders, Behavioural and psychological symptoms of dementia, Quality of life

## Abstract

**Background:**

Alzheimer’s disease and related disorders are characterized by cognitive impairment associated with behavioral and psychological symptoms of dementia. These symptoms have significant consequences for both the patient and his family environment. While risk factors for behavioral disorders have been identified in several studies, few studies have focused on the evolution of these disorders. Moreover, it is important to identify factors linked to the long-term evolution of behavioral disorders, as well as patients’ and caregivers’ quality of life. Our purpose is to present the methodology of the EVITAL study, which primary objective is to determine the factors associated with the evolution of behavioral disorders among patients with Alzheimer’s disease and related disorders during the year following their hospitalization in cognitive and behavioral units. Secondary objectives were 1) to assess the factors related to the evolution of behavioral disorders during hospitalization in cognitive and behavioral units; 2) to identify the factors linked to patients’ and caregivers’ quality of life, as well as caregivers’ burden; 3) to assess the factors associated with rehospitalization of the patients for behavioral disorders in the year following their hospitalization in cognitive and behavioral units.

**Method/Design:**

A multicenter, prospective cohort of patients with Alzheimer’s disease and related disorders as well as behavioral disorders who are hospitalized in cognitive and behavioral units.

The patients will be included in the study for a period of 24 months and followed-up for 12 months. Socio-demographic and environmental data, behavioral disorders, medications, patients’ and caregivers’ quality of life as well as caregivers’ burden will be assessed throughout hospitalization in cognitive and behavioral units. Follow-up will be performed at months 3, 6 and 12 after hospitalization. Socio-demographic and environmental data, behavioral disorders, medications, patients’ and caregivers’ quality of life, unplanned rehospitalization as well as caregivers’ burden will also be assessed at each follow-up interview.

**Discussion:**

The present study should help better identify the factors associated with reduction or stabilization of the behavioral and psychological symptoms of dementia in patients with Alzheimer’s disease. It could therefore help clinicians to better manage these symptoms.

**Trial registration:**

Clinical Trials NCT01901263. Registered July 9, 2013.

## Background

Alzheimer’s disease and related disorders (ADRD) are characterized by cognitive impairment associated with behavioral and psychological symptoms of dementia (BPSD) [1]. The BPSD are common in ADRD and often associated with one another [2-4]. They may occur at all stages of the disease and worsen with cognitive decline.

Besides apathy and depression, which are frequent during the early/middle stages of the disease (40 to 60% of patients), other BPSD such as agitation (30%), irritability (30%), delusions (10 to 20%), aberrant motor behavior (15 to 30%) and hallucinations (6 to 10%) [5,6] can be very upsetting for both the patient and the caregiver.

BPSD have significant consequences for both the patient and the family as they may lead to difficulties in activities of daily living (ADL) [3] and interfere with patients’ quality of life (QoL) [7]. Moreover, they are associated with faster cognitive impairment [8]. They increase the risk of hospitalization as well as developing other complications of dementia. Furthermore, they can lead to an increase in caregivers’ health problems and a decrease in their QoL [7]. Studies have shown that the severity of BPSD was connected with the burden and psychological state of caregivers [9-11]. All these consequences result in increased health expenditures [12]. Finally, they can precipitate nursing home placement regardless of cognitive impairment [13,14].

Identification of BPSD is important to optimize ADRD patient management. Current treatment strategies consist in non-drug therapies and subsequently pharmacological treatments, including specific treatment for Alzheimer’s disease. A previous study showed that the management of BPSD can reduce the rate of home nursing placement as well as caregivers’ burden and increase a QoL [15]. The unpredictable occurrence of these symptoms and difficulty to stabilize them may lead to crises. The French Alzheimer Plan 2008–2012 has provided specific hospital units called cognitive and behavioral units (CBUs). These units are intended for patients with ADRD and productive behavior disorders. While risk factors for BPSD have been identified in several studies, few studies have focused on the evolution of these symptoms. To our knowledge, only two studies focused on BPSD evolution in patients admitted to CBUs. The first one assessed short-term BPSD evolution from CBU admission to discharge [16]. The second showed a favorable effect of hospitalization in the CBU on BPSD at hospital discharge and 15 days after discharge [17]. Beyond the assessment of the short-term BPSD evolution during hospitalization in CBUs, it would be important to identify factors that are linked to the long-term evolution of BPSD, as well as patients’ and caregivers’ QoL. The EVITAL study was designed to assess these questions. The purpose of this paper is to provide the methodology of the EVITAL study.

### Study objectives

#### Primary objective

- To identify the factors associated with the long-term evolution of BPSD among patients with ADRD during the year following their hospitalization in the CBU.

#### Secondary objectives

- To assess the factors related to the reduction of BPSD during hospitalization in CBUs.
- To identify the factors linked to patients’ and caregivers’ QoL, and caregivers’ burden.
- To assess the factors associated with rehospitalization of the patients for BPSD in the year following their hospitalization in CBUs.

## Methods/Design

### Study design

This is a multicenter prospective cohort study.

### Setting and patients

The EVITAL study will take place in the CBUs of 3 different hospitals located in Lyon and Saint Etienne, France.

Inclusion criteria are: age 50 years or older, admission to the CBU of participating centers, ADRD patients at the dementia stage, no severe progressive or unstable disease that may interfere with the results of the assessment, and presence of a caregiver having sufficient contact with the subject to be able to notice behavioral changes.

Exclusion criteria are: patients at high risk of death and isolated patients.

### Ethics approval

The study protocol has been reviewed and approved by an Ethics Committee (Comité de Protection des Personnes). This committee covers the ethical approval of all three sites of our data collection. All procedures are in accordance with the Declaration of Helsinki and the International Conference on Harmonization (ICH) Good Clinical Practice Guidelines.

This is a routine care research that does not require written informed consent. An information sheet will be given to the patient and his/her caregiver at admission in the CBU.

Their acceptance or refusal will be noted in medical records.

### Independent variables

#### Clinical and neuropsychological assessment

An interview and a physical examination will be performed by the neurologist, geriatrician or psychiatrist in charge of the patient. Other factors favoring BPSD such as family situation, living conditions, previous diseases, comorbidities, date of diagnosis, and medications will be investigated during hospitalization.

For patients living at home, relationship with informal and professional caregivers will be collected.

Global cognitive function will be assessed using the Mini Mental State Examination (MMSE) [18], with scores ranging from 0 to 30 points.

#### Hospitalization in CBUs

The following factors will be collected during hospitalization in the CBU are: reason for hospitalization, length of stay, type of non-drug therapies used.

#### Use of psychotropic drugs

Psychotropic drug use will be collected at CBU admission and discharge, and at each follow-up interview.

### Dependent variables

#### Evaluation of BPSD

Among the scales used for exploring these disorders, the Neuropsychiatric Inventory (NPI) [19] is commonly used. It is often used in international studies and has been validated in French [20].

The NPI is a questionnaire designed to collect information on neuropsychiatric problems from patients with brain disorders.

Behavioral disorders will be assessed during the initial visit and each subsequent interview with the patient and the caregiver. The short form of the Neuropsychiatric Inventory (NPI-Q) will be used. Twelve types of neuropsychiatric symptoms are covered by the questionnaire: delusions, hallucinations, agitation, depression, anxiety, euphoria, apathy, impulsive behavior, mood swings, aberrant motor behavior, and eating and sleeping disorders. For each symptom, the severity, frequency and effect on the caregiver are assessed. A total subscale score is calculated by multiplying frequency and severity, and a global score is generated by summing up the total scores of the individual subscales (maximum = 144). Score measuring the effect on the caregiver ranges from 0 to 60 points.

The “family” NPI will be given to the caregiver at home or referent caregivers at the nursing homes. The “healthcare team” NPI will be completed by the healthcare team during hospitalization.

#### Patients’ quality of life

The EuroQuol EQ-5D is used for assessing the QoL of patients with Alzheimer’s disease. This scale includes five areas: mobility, self-care, daily activity, physical pain, and anxiety/depressive symptoms. A one-to-five scale is used for each dimension. The scale will be completed by both patient and caregiver about their own QoL. In addition, the caregiver will answer the questions about the patient’s QoL [21].

#### Caregivers’ quality of life and caregivers’ burden

The Caregiver’s Quality of Life (French abbreviation QDVA) is a 20-item scale exploring caregiver’s QoL. Four categories of situations are studied, according to the angst and coping capability of caregivers. Caregiver’s QoL and vulnerability are assessed, taking into account the socio-demographic characteristics of both patients and their principal caregivers, and the patients’ medical and therapeutic data [22]. Each item is coded 5 (high QoL) or 0 (weak QoL). A total scale score represents the percentage of remaining QoL.

The subjective caregivers burden will be assessed using the mini-Zarit, a validated short version of the Zarit Burden Inventory, which was previously developed for routine medical care [23].

#### Unplanned hospitalization

The duration and reason for unplanned hospital readmission will be noted during the year following discharge.

### Data collection procedure

**Figure 1:**
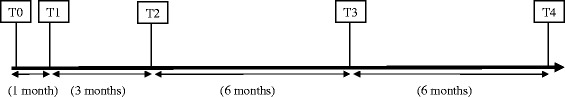
**Typical schedule for a patient included in the EVITAL protocol: a summary of different stages.** T0 = inclusion visit. T1 = End of hospitalization visit. T2 = 3-month follow-up visit at home. T3 = 6-month follow-up phone call. T4 = 12-month follow-up phone call.

Typical schedule for a patient included in the EVITAL protocol is given in Figure 1.

#### Inclusion visit

The inclusion visit will take place on the day of patient admission in the CBU. It will be carried out by the neurologist, geriatrician or psychiatrist in charge of the patient. Inclusion criteria will be validated through an interview with the patient and his/her caregiver. During this examination, various data will be collected: demographic and environmental data, patient’s living conditions, previous diseases and comorbidities, medications and non-drug therapies, global cognitive performance (MMSE), BPSD (NPI and NPI-ES), caregivers’ burden (mini-Zarit), and patients’ and caregivers’ QoL (EQ-5D, QDVA).

#### End of hospitalization visit

This visit will be carried out by the neurologist, geriatrician or psychiatrist in charge of the patient according to standard practices. During this visit, the following data will be collected: demographic and environmental data, medications and nondrug therapies used duringhospitalization, and BPSD at the outcome (NPI-ES).

#### Follow-up

A follow-up visit will be performed 3 months after discharge at the patient’s residence (home or nursing home). Follow-up phone calls will be made to caregivers (patients’ informal caregivers at home or referent caregivers at the nursing homes) at 6 and 12 months. During these follow-up interviews, the following data will be collected: demographic and environmental data, patient’s living conditions, concomitant events, medications and non-drug therapies, BPSD (NPI and NPI-ES), caregivers’ burden (mini-Zarit), and patients’ and caregivers’ QoL (EQ-5D, QDVA).

### Sample size

As this is a prospective, observational cohort study that will includes a large number of specific factors to be analyzed, there is no issue of statistical power to be considered. However, we need a sufficient number of participants to obtain useful outcome measures. The three participant CBUs have a total of 300 patients per year. We can expect that about 60% of patients will consent to participate and meet the inclusion criteria. Preliminary analysis made on the 50 first patients gave an annual attrition rate of 20% during the 12-month follow-up period, mainly due to death. All patients who meet the inclusion criteria and are hospitalized in the 3 participant CBUs in the span of two years will be included in the study.

### Data analysis

Univariate analysis will be first performed to assess each study objective. Associations between the rehospitalization or psychotropic consumption rates and independent qualitative variables will be determined with chi2 tests. The link between the NPI score and qualitative risk factors will be assessed using Student t tests. Multivariate analyses will be used to take into account potential confounding factors.

To identify the factors associated with the evolution of BPSD in patients with ADRD during the year following their hospitalization in the CBU, linear regression models and survival models will be used.

The link between management in CBUs and evolution of BPSD during patients’ hospitalization will be assessed with linear regression models and survival models.

To identify the factors associated with patients’ and caregivers’ QoL, and caregivers’ burden, linear regression models and survival models will be used.

The association between changes in psychotropic drug consumption (especially neuroleptic drugs) and the evolution of BPSD will be assessed with linear regression models and survival models.

Finally, to assess the factors associated with rehospitalization of the patients for BPSD in the year following their hospitalization in the CBU, logistic regression models will be used.

SPSS software will be used for data analyzes.

## Discussion

The EVITAL project aims to better understand the factors associated with the evolution of BPSD in the year following patient hospitalization in CBUs. It should help us better identify the factors affecting patient’s and caregiver’s QoL. Moreover, it could bring out factors that modulate the risk of unplanned hospital readmission. Finally, it should contribute to a better understanding of the natural history of BPSD.

The originality of this project is based on three criteria: the choice of the study population, duration of follow-up and inclusion of patients from different CBUs. Our study focuses on patients with ADRD and productive BPSD requiring hospitalization in a specific unit. To our knowledge, only two previous studies assessed the evolution of BPSD during hospitalization in CBUs. The results of these studies were encouraging, showing a decrease in BPSD after patient management in CBUs. However, both studies were limited as they only assessed the evolution of the BPSD from patient admission to discharge [16] and 15 days after discharge [17], respectively. These studies did not give us information on the long-term outcome of the patients after CBU management. The present study is the first to follow patients for one year, with assessments performed 3, 6 and 12 months after their CBU discharge.

Moreover, these studies were conducted in only one CBU whereas the present study includes patients from different CBUs. The multicenter design may allow us to include patients with various profiles, regardless of each unit specific organization.

BPSD are also known to have a negative impact on both patients’ and caregivers’ quality of life [7], which is an important point to consider. Moreover, caregivers play a major role in helping patients stay at home, which is one of the CBU objectives. It was showed that successful patient management with a specific pharmacological or non- drug therapy can have a positive impact on both patients’ and caregivers’ QoL, which are intrinsically connected to each other [24]. It will be interesting to observe the evolution of their QoL as well as caregivers’ burden after CBU management.

The assessment of patients admitted to CBUs will help better describe and value the work done by the CBU healthcare team. Moreover, the results of this study could be helpful for other CBU teams to develop effective management strategies in the CBU, and to better identify patients at high risk of increasing BPSD. The database designed in the present research could also be proposed to other CBUs to help them better describe their activity with standardized data.

The EVITAL study may have several limitations. Some patients who were living at home before their admission in the CBU may move to a new place of residence, e.g. in an institution, after discharge. Some scales, such as the mini-Zarit, will be therefore used only for patients living at home. Furthermore, the NPI will be assessed by two different persons. However, the inter-rater reliability of the NPI has been established [19] and the number of patients involved should be limited, as one of the main objectives of CBU management is to facilitate the return to their initial place of residence after discharge.

In spite of these limitations, the management of patients with Alzheimer’s disease or related diseases is currently a major challenge. Therefore, it seems important to better identify the factors associated with BPSD reduction and patients’ and caregivers’ QoL in order to improve patient management in the CBU and after hospitalization discharge. The EVITAL project will help better understand these topics.
